# Emerging Understanding of Multiscale Tumor Heterogeneity

**DOI:** 10.3389/fonc.2014.00366

**Published:** 2014-12-18

**Authors:** Michael J. Gerdes, Anup Sood, Christopher Sevinsky, Andrew D. Pris, Maria I. Zavodszky, Fiona Ginty

**Affiliations:** ^1^Diagnostic Imaging and Biomedical Technologies, GE Global Research, Niskayuna, NY, USA

**Keywords:** cancer, heterogeneity, tumor microenvironment, multiplexing, tumor mechanisms, multi-omic analysis, next-generation sequencing

## Abstract

Cancer is a multifaceted disease characterized by heterogeneous genetic alterations and cellular metabolism, at the organ, tissue, and cellular level. Key features of cancer heterogeneity are summarized by 10 acquired capabilities, which govern malignant transformation and progression of invasive tumors. The relative contribution of these hallmark features to the disease process varies between cancers. At the DNA and cellular level, germ-line and somatic gene mutations are found across all cancer types, causing abnormal protein production, cell behavior, and growth. The tumor microenvironment and its individual components (immune cells, fibroblasts, collagen, and blood vessels) can also facilitate or restrict tumor growth and metastasis. Oncology research is currently in the midst of a tremendous surge of comprehension of these disease mechanisms. This will lead not only to novel drug targets but also to new challenges in drug discovery. Integrated, multi-omic, multiplexed technologies are essential tools in the quest to understand all of the various cellular changes involved in tumorigenesis. This review examines features of cancer heterogeneity and discusses how multiplexed technologies can facilitate a more comprehensive understanding of these features.

## Introduction

Cancer can be seen as the summation of many different cell types and is best described by the hallmarks of cancer (1, 2). To date, 10 hallmarks have been described: self-sufficiency in growth signals; insensitivity to antigrowth signal; tissue invasion and metastasis; unlimited proliferation potential; sustained angiogenesis; evading apoptosis; deregulated metabolism; genomic instability; tumor promoting inflammation; and avoiding immune destruction (1, 2). These acquired capabilities may vary across individuals, organ systems, subtypes within an organ, and cancer stage.

**Figure 1:**
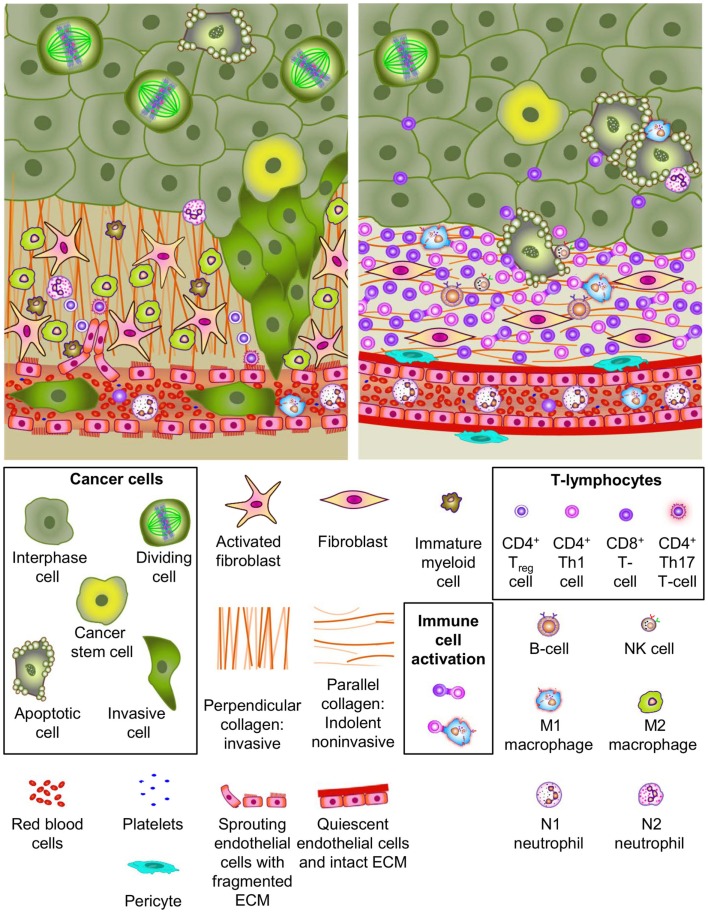
**Cellular heterogeneity in the tumor and microenvironment**. Most solid tumors grow in a complex micro-environment consisting of stromal cells, vasculature, infiltrating immune cells, and complex extracellular matrix (ECM) components. Cell types and ECM components are identified in the legend. The upper left panel depicts many elements reported in association with tumor promoting micreoenvironments. This environment exhibits tumor promoting characteristics including (1) paracrine signaling axes between tumor cells, stromal cells, vascular cells and immune cells, (2) neoangiogeneis with porous/leaky vascular ECM, (3) reactive stroma, (4) ECM remodeling, and (5) tumor cell invasion and intravasation. Notable tumor promoting immune-cell phenotypes are highlighted. Many of these factors have been demonstrated to contribute to invasive growth and metastatic dissemination of cancer cells. The upper right panel illustrates tumor micro-environment characteristics reported to be associated with a more indolent phenotype. Several important characteristics of indolent tumors including (1) innate immune-cell mediated tumor cell killing, (2) cellular and humoral adaptive immune-cell anti-tumor responses, (3) normal vasculature with pericyte coverage and intact basement membrane, (4) quiescent stroma, and (5) parallel collagen orientation are shown.

The hallmarks of cancer are driven by acquired intra- and intertumoral genetic and epigenetic variations. Intertumoral heterogeneity has resulted in the classification of discrete tumor subtypes, which are characterized by distinct molecular genetic profiles, morphology, and expression of specific markers. Intratumoral heterogeneity manifests as variations within the tumors, including cells adopting a range of functional properties and different biomarker expression patterns. The tumor microenvironment and its related cell types also contribute to malignant transformation. Figure 1 illustrates the cellular milieu and interactions within the tumor and surrounding microenvironment, including immune-cell interplay. This will be further discussed later in the review. Furthermore, the hereditary genetic baseline of each individual can modify overall physiology, drug uptake, metabolism, and half-life/clearance leading to outcome variations. Similarly, differences in the innate and adaptive immunity and DNA damage responses play critical roles. The combination of all of these factors results in a highly complex and multifaceted disease state.

Understanding the interplay between the different elements and their roles in tumor progression and treatment response is a challenging, but important, consideration. It is of particular relevance when developing novel drugs, in understanding how drug resistance develops, and when directing patients toward effective secondary therapies. The growing appreciation of cancer complexity has been accompanied by the recognition that tools and technologies used historically in drug discovery and cancer diagnosis are limited in their ability to fully elucidate mechanisms and pathways at single cell, multicellular, and system level. Consequently, the field is transitioning toward platforms that encompass multiplexed, multi-omic, and computational technologies. This review discusses cellular heterogeneity within tumors and considers how novel technologies are providing new approaches to cancer research and biomarker identification. Heterogeneity at the level of cancer progression and tumor evolution is considered first, followed by a discussion on the observed diversity at the histological and molecular level. The focus then switches to the differences in cell signaling and the importance of the tumor microenvironment in tumorigenesis. In parallel, examples of current and emerging methods and technologies that are being used in cancer research and diagnosis are also highlighted and discussed.

## Heterogeneity in Clonal Evolution during Tumor Progression

Cancer arises as a consequence of genetic mutations (3) and epigenetic alterations (4) within developing neoplastic cells. Currently, there are two theories that describe the establishment and maintenance of tumors: clonal evolution and the stem cell hypothesis. The clonal evolution model is based on the premise that over time, cancers continue to evolve by virtue of a Darwinian process of genetic drift and natural selection. Genetic instability within the tumor cell population leads to accumulation of additional mutations within single cells. Thus, a number of genetically divergent clonal subpopulations exist, with the most aggressive cells driving tumor progression (3, 5–9).

The stem cell hypothesis suggests that only a subset of cancer cells, defined as cancer stem cells, can participate in “clonal” evolution (10–13) and drive tumor progression, while the other cells are “evolutionary dead ends” (12, 13). The resulting hierarchical organization consists of stem cells, intermediate progenitors, and terminally differentiated progeny. Cells arising from different cell types will produce tumors of vastly different phenotypes and biology (14, 15). This has been demonstrated in several inducible colon tumor models in which the tumor-suppressing APC protein was selectively ablated in either an active (LGR5) or quiescent (Lrig1) stem cell population in the gut. When tumorigenesis was activated in the LGR5 population, localization of tumors was restricted to the upper gastrointestinal tract (16) while those originating from the Lrig1 population developed in the distal colon (17). The underlying mechanisms for this localized tumorigenesis remain enigmatic as both stem cell types are found in the crypts throughout the gastrointestinal tract. Furthermore, evidence from cancer cell transplantation experiments have established that different malignancies exhibit a broad spectrum of stem cell frequencies [reviewed by Visvader and Lindeman (18)] and different tumors vary in their cancer stem cell composition (13).

Cancer stem cells have aroused interest as therapeutic targets because of their purported role in tumorigenesis and metastasis (10, 19–21) and contribution to chemoresistance (22–24). *In vitro* assays have demonstrated that there are distinct populations of tumorigenic and non-tumorigenic cells in various cancers, including breast and colorectal (25, 26), and studies in transgenic models have shown that tumors arising from stem cells establish more readily and are more aggressive (27–29). Cancer stem cells may also contribute to drug resistance and disease recurrence through expression of multidrug resistance proteins, including ABCB1, ABCG2, and ABCB5 (30).

**Figure 2:**
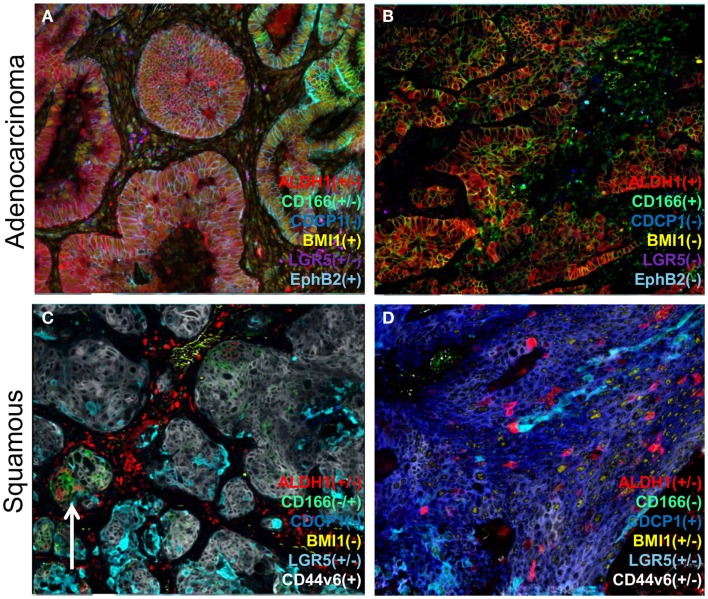
**A representation of heterogeneity in cancer stem cell marker expression**. A series of lung cancers were examined for the expression of various reported cancer stem cell markers using a multiplexed protocol on the MultiOmyx™ platform to illustrate the heterogeneity in cancer stem cell protein markers. Examples of adenocarcinoma **(A,B)** show two different cellular profiles with the sample on the left **(A)** displaying intratumoral heterogeneity for ALDH1 and CD166 (compare left and right sides), whereas another sample **(B)** was homogenous for two of the markers), and devoid of BMI1, LGR5, and EphB2. Different profiles were found in squamous carcinoma samples **(C,D)** with CD44v6 (gray/white) expression found in both samples, and on the right **(D)** was accompanied by CDCP1. ALDH1 was only found in a minor population of tumor cells in both samples, and on the left **(C)** was uniquely associated with CD166 minor (arrow) clusters of cells.

While proteomics and genomics methods have been widely used to elucidate stem cell biology, identification of cancer stem cells *in situ* using immunofluorescence methods allows direct assessment of heterogeneity, cell types, and numbers. Typically, a number of cell-specific protein markers are needed for such cell characterization. Some markers, such as ALDH1, CD133, and CD44, are common across all tumors, while others may be relatively tumor specific, e.g., CD271 in melanoma and Trop2 for prostate (30). Even within the same cancer type, the cell markers vary depending upon the different histologic/molecular subtype. For example, in non-small cell lung cancer (NSCLC), variations in the expression of stem cell markers have been observed between adenocarcinoma and squamous cell cancers using multiplexed immunofluorescence, with similar complexity seen in either of the two tumor subtypes (representative example shown in Figure 2). The significance of such stem cell diversity in terms of patient outcome or drug response remains to be determined. In a recent study where multiple markers were examined in breast cancer cell lines and primary tumors, little concordance was seen in co-expression of the markers with clinical responses (31). Conversely, in another study where three breast stem cell markers (CD24, CD44, and ALDH1) were examined, expression patterns were found to correlate to histopathological subtype of the tumors (32). Moreover, tumor subtype has been shown to influence the local stem cell populations in adjacent normal epithelia in breast cancer, where triple-negative tumors contained CD44^+^CD49f^+^CD133/2^+^ stem cells in nine out of nine samples, while in estrogen receptor (ER)-positive tumors, this was detected in only 7 out of 52 samples examined (33).

## Histological and Molecular Heterogeneity

Histological assessment is the most common means of distinguishing cancer from benign tissues and identifying the subtype. Molecular subtyping characterizes an additional layer of heterogeneity by establishing the predominant genomic and protein signatures present. This is often found to be complementary to traditional histological classification, wherein a single histological type may be divided into discreet molecular subtypes. Breast and lung subtypes have been studied extensively, and there is an emerging understanding of colorectal cancer subtypes. In addition, it has been suggested that other cancers, such as gastric (34), prostate (35), and ovarian (36), may also exhibit different molecular subtypes. As will be elaborated on below, the need for multiple markers to distinguish histologic and molecular subtypes is currently enabled by singleplex immunohistochemistry (IHC) and multiplexed gene-expression assays. These examples represent just a brief summary of the biological complexity and range of diagnostic testing for three major cancer types. The transition from research biomarker to prognostic or predictive diagnostic test can involve years of research, biomarker down-selection, verification, and clinical validation. Successful translation is highly dependent on a number of key variables including sample collection, quality, technical performance of the analytical platform, and validation in adequately powered, clinically relevant patient populations (37).

### Breast cancer

Five intrinsic molecular subtypes have been identified for breast cancer: luminal A, luminal B, human epidermal growth factor receptor 2 (EGFR2 or HER2)-positive, triple-negative, and normal-like (38). The subtypes partially reflect clinical phenotypes based on the presence or absence of the ER, progesterone receptor (PgR), and HER2 (39) and each is associated with a distinct prognosis and clinical outcome. In addition to providing classification information, both the ER and the PgR expression (typically determined by IHC) are valid prognostic and predictive markers in the adjuvant and metastatic settings. ER is a predictor of a positive response to endocrine therapy, although not all patients with ER-positive disease benefit from endocrine therapy, and guidelines recommend that tumor PgR status is also evaluated (40). Patients with a positive status for either or both of the receptors typically receive endocrine therapy (41). *HER2* overexpression, as determined by IHC and/or FISH amplification is generally associated with poorer prognosis and is also used as a predictive marker for trastuzumab/HER2-directed therapy (42). Patients negative for all three markers are referred to as having triple-negative breast cancer (TNBC). More recently, additional TNBC subtypes have been described including the “molecular apocrine” group, which is related to activation of the androgen receptor (43), the “interferon” subtype (44), and the “claudin-low” subgroup (45). Neoadjuvant or adjuvant chemotherapy regimens are standard of care for TNBC patients but as yet there are no approved predictive biomarkers of therapy response.

The proliferative marker Ki67 may also be useful as a prognostic indicator for breast cancer patients (46). Ki67 expression by IHC has been shown to correlate with overall survival and disease-free survival, with high levels of Ki67 indicative of an increased risk of recurrence (47). However, controversy remains over the criteria for defining tumor positivity. Moreover, clinical utility has been hampered by preanalytical, analytical, and scoring variability, although recent efforts have been made to address this (48).

In addition to standard of care testing for ER, PgR, and HER2, there are several multimarker tests now available for breast cancer outcome including: MammaPrint^®^ (49) (Agendia, Irvine, CA, USA); a five-antibody IHC panel Mammostrat^®^ (50) (Clarient Diagnostic Services Inc., Aliso Viejo, CA, USA); Onco*type* Dx^®^ (51) (Genomic Health, Redwood City, CA, USA); the PAM50-based Prosigna™ assay (52) (NanoString Technologies, Seattle, WA, USA). Each of these has proven clinical utility for predicting recurrence in patients with ER-positive, node-negative breast cancer. Onco*type* Dx has also been shown to predict chemotherapy benefit in the high-risk patient group, with minimal benefit in the low-risk group (53). Consequently, based on the risk of recurrence score, the test helps physicians determine who is likely to benefit from adjuvant systemic chemotherapy. Clinical studies have shown that over 30% of treatment recommendations changed based on the patient risk score, and this has led to a net reduction in chemotherapy use (54). As more therapies and companion diagnostic biomarkers become validated, the need for multiple gene and protein biomarkers is likely to increase.

### Lung cancer

Lung cancer is also comprised of two major subtypes: small cell lung cancer (SCLC) and NSCLC. NSCLC can be divided histologically into adenocarcinoma, squamous cell, and large cell lung carcinoma (55). The clinical significance of accurate subtyping is demonstrated with bevacizumab, which is contraindicated in patients with squamous cell NSCLC (56) due to the elevated risk of life-threatening hemorrhage (57). Indeed, commercial tests are now available to distinguish between adenocarcinoma and squamous cell NSCLC. Examples include the ProOnc Squamous Dx (Prometheus Laboratories Inc., San Diego, CA, USA) based on quantitative expression of micro RNA miR-205 (58), and the Pulmotype^®^ test (Clarient Diagnostic Services Inc.), which uses a panel of five IHC markers (cytokeratin 5/6, MUC-1, TRIM-29, CEACAM-5, SLC7A5) to aid in distinguishing subtypes (59). InCyte Diagnostics (Spokane Valley, WA, USA) also offers a panel of IHC markers including thyroid transcription factor-1 (TTF-1), napsin A, cytokeratin 5, cytokeratin 7, and p63; a positive stain for TTF-1 and napsin A supports a diagnosis of adenocarcinoma, while the others indicate tumors of a squamous subtype.

Non-small cell lung cancer can also be defined by different molecular subtypes based on mutations within driver oncogenes. The three most established biomarkers are *EGFR* mutations, echinoderm microtubule-associated protein-like 4 (*EML4)-*anaplastic lymphoma kinase *(ALK)* rearrangements, and *KRAS* mutations. Each has been shown to have prognostic and predictive value. For example, patients whose lung tumors harbor *EGFR* exon 19 deletions or exon 21 (L858R) substitutions are now offered one of two tyrosine kinase inhibitors, erlotinib or afatinib, as a first-line treatment (39). However, in those patients with the *EML4-ALK* fusion gene [found in 2–7% of NSCLC patients (60)] crizotinib is the primary first-line treatment option (61). Routine testing for both *EGFR* mutations and *ALK* fusions is now recommended by the College of American Pathologists and the International Association for the Study of Lung Cancer (62). Other biomarkers are also being studied for potential utility in guiding NSCLC treatment decisions. These include PIK3CA, HER2, BRAF, ROS, RET, NRAS, MET, and MEK1. The onerous demands of molecular and protein analysis on biopsied tumor material may limit the number of tests that can be conducted.

### Colorectal cancer

Colorectal cancer has also been shown to comprise clinically distinct molecular subtypes, although the exact number is currently unclear. A recent study (63) defined six clinically relevant subtypes, each of which is similar to normal colon crypt cells but with varying degrees of stemness and Wnt signaling. Other reports have identified three subtypes based on genomic characteristics: the chromosomal-unstable, the microsatellite-unstable CpG island methylator phenotype (64), and a third subtype, which is largely microsatellite and chromosomally stable (65). Roepman et al. (66) also identified and validated three colorectal cancer subtypes: mismatch repair deficient epithelial, proliferative epithelial, and mesenchymal; with each subtype potentially having a different therapy response. In addition to these findings, a recent proteomics analysis has further refined colorectal cancer classification. The proteomes of 90 patient samples characterized previously by the Cancer Genome Atlas were analyzed by mass spectrometry (MS), and five subtypes were proposed based on consensus cluster analysis (67). Aside from analyzing correlations between genomic and proteomic features, the authors examined classification agreement between three genomics-based classifiers and the proteomic subtypes. Despite some overall consistency, the classifiers exhibited considerable differences in assigning patients to subtypes. This suggests that further analyses and categorization approaches may yield better disease classification.

Other biomarkers used to help treatment decisions in colorectal cancer include the use of *KRAS* mutation status as a predictive marker (68) and the use of *BRAF* mutations as a strong negative prognostic indicator in *KRAS* wild-type colorectal cancer (69).

## Cell Signaling and Heterogeneity

**Figure 3:**
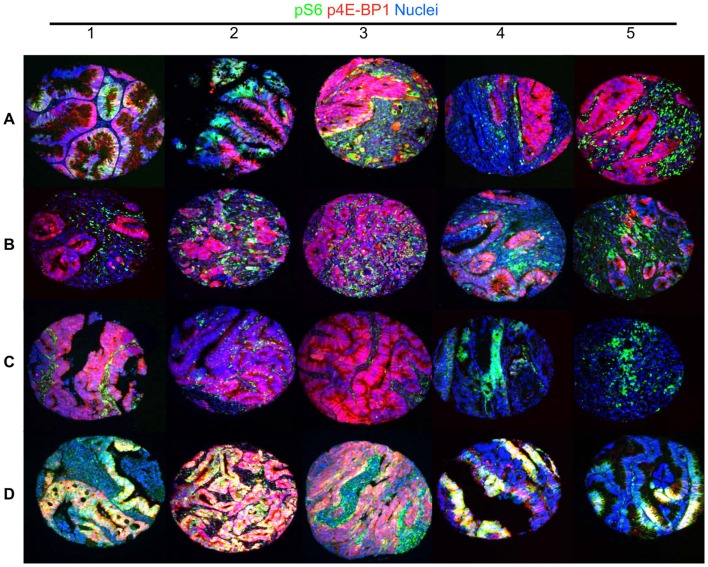
**A representative image of heterogeneity at the cell signaling level in colorectal cancer**. mTOR targets phospho-Ribosomal Protein S6 and phospho-4E-BP1 exhibit substantial cellular exclusivity in colorectal cancer specimens, with rare coexpression (representative of previously published results (71). This figure shows 20 TMA cores containing 2,000–5,000 cells, which have been generated to demonstrate the heterogeneity of cell signaling in colorectal cancer. Rows **(A–C)** show different patterns of mutually exclusive p4E-BP1 (red) and pS6 (green) signals. Many specimens exhibit a strong signal for each phosphorylation event in exclusive tumor cells **(A1–A3)**. Another notable pattern exhibits substantial p4E-BP1 expression in tumor cells, and a high level pS6 signal in stromal cells **(A4–C2)**. In addition, rare tumors exhibit high level signal in only one of the canonical mTORC1 substrates **(C3–C5)**, or both simultaneously **(D1–D5)**.

Understanding cellular and molecular differences within cancer subtypes is critical for understanding disease heterogeneity (70). With that, there has been a drive to develop and adopt new technologies capable of single-cell analysis and measurement of activated pathways and other genomic aberrations. MultiOmyx™(GE Healthcare, Aliso Viejo, CA, USA) is one such technology that provides multiplexed protein analysis and DNA alterations (by FISH) to be imaged and quantified within the same cells of intact single fixed tissue section (71). Using this technology, a study of formalin-fixed paraffin-embedded (FFPE) specimens from a cohort of over 700 colorectal cancer patients demonstrated considerable differences in the phosphorylation of two proteins, ribosomal protein S6 (S6) and eukaryotic initiation factor 4E binding protein 1 (4E-BP1), in individual cells within tissue microarray tissue cores (71). Mutually exclusive phosphorylation patterns of these two canonical substrates of mammalian target of rapamycin complex 1 (mTORC1) were observed in individual cells, in large regions of most tumors, and in distinct cell lineages (representative images shown in Figure 3), thus demonstrating differential pathway activation. Mutual exclusivity of pathway activation was seen in tumor regions typically consisting of over 2,000 cells, and in cells adjacent to one another (Figure 3). Single cell, cluster, and heat map analyses were used to quantify and visualize the heterogeneity of the mTOR signaling dynamics in this disease state (71).

Other examples of signaling heterogeneity have been shown using multispectral imaging of several proteins from a common signal transduction pathway. For example, multispectral imaging was used to demonstrate that activation of the c-MET signaling pathway and consequent induction of epithelial–mesenchyme transition are common features in prostate cancer (72). Another technology, reverse-phase protein array (RPPA), has been used for the quantitative analysis of proteins in their phosphorylated or unphosphorylated forms in arrays of cell lysates, plasma, or serum samples. Its use in research and clinical settings has recently been extensively reviewed by Gallagher and Espina (73). The Collaborative Enzyme Enhanced Reactive-immunoassay (CEER) (74) is another platform that can be used to detect protein expression and phosphorylation at the single-cell level. By way of example, CEER has been used to identify heterogeneity in activated signaling pathways in advanced gastric cancers (75).

In summary, elucidation of the cancer heterogeneity and its clinical relevance requires multiple approaches, including histological and subtype analysis, cell composition and distribution, genomic alterations and *in situ*, and extracted protein and gene expression. Depending on the question and desired granularity, some or all of these technical approaches may be required. The amount of available sample, its age and state of preservation, and ability to do potentially complex multi-omic analysis also need to be considered.

## Tumor Microenvironment – Heterogeneity and Anticancer Therapeutic Target

Aside from the malignant cells themselves, the tumor microenvironment is known to play a vital role in tumorigenesis as its constituent cells and structures affect how tumor cells grow and spread (76). The tumor microenvironment is a highly heterogeneous mix of cellular and non-cellular components, consisting of the extracellular matrix (ECM), vasculature, fibroblasts, smooth muscle cells, immune cells, nerves, and proteins in the immediate extracellular environment (77). There are at least three distinct processes through which the tumor micro-environment promotes tumor growth: stromal cell secretion of paracrine-acting stimulatory factors; angiogenesis; and immune-mediated interactions. These processes are interconnected and work together to produce a morphological and chemical micro-environment wherein tumor cells thrive due to the ready supply of growth factors, cytokines, and vasculature (78). Conversely, some tumors are characterized by a tumor antagonistic microenvironment. Both scenarios are illustrated in Figure 1. Ongoing efforts to characterize the cells and tissues of the tumor microenvironment is expected to reveal additional insights into the mechanisms of tumor progression and metastasis. These gains should ultimately influence cancer diagnosis and therapy.

### Extracellular matrix

The ECM is an important component of the microenvironment and serves as the substrate for cell adhesion and in local growth factor regulation. The architecture of the ECM is altered during carcinogenesis, and its remodeling is believed to be crucial for tumor malignancy and metastatic progression (79, 80). For example, in normal breast tissue, collagen I fibrils are relaxed, and non-oriented. However, in breast cancer, collagen I is often highly linearized and oriented next to the epithelium or projecting perpendicularly into the tissues (Figure 1) (81). Moreover, breast cancer growth can be selectively accelerated or slowed by increasing or decreasing ECM crosslinking, and progression is accompanied by ongoing increases in ECM stiffness (81). The ECM also has a key role in disease progression in pancreatic cancer, where the abundance of ECM induces an abnormal configuration of blood and lymphatic vessels. The rigidity of the ECM compresses blood vessels leading to reduced perfusion, which is proposed to impede the delivery of drugs to neoplastic cells and contribute to drug resistance (82). Recent *in vitro* and *in vivo* mechanistic studies have pointed to a tripartite cellular interaction with the ECM, whereby tumor cells and macrophages migrate to endothelial cells by trafficking along collagen fibrils with specific structural properties (83). These observations underscore the complexities of cooperativity between cells and associated structures in the tumor microenvironment.

### Angiogenesis

Angiogenesis has long been recognized as playing an important role in tumor formation. During tumorigenesis, the appropriate balance between proangiogenic and antiangiogenic molecules and autocrine and paracrine growth factor stimulation is lost (84). The main mechanism, known as endothelial sprouting, depends on vascular endothelial growth factor upregulation and the development of functional interactions between endothelial cells, pericytes, stromal cells, and the associated ECM (Figure 1) (85, 86). As angiogenesis is critical for tumor survival, it is a natural and now well-established target for therapeutic intervention. Modest improvements in survival are seen on inhibiting angiogenesis in some cancers suggesting that additional angiogenesis-promoting targets alone, or in combination with other targeted therapies, may yield improved response rates (87, 88).

### Immune cells

The tumor microenvironment also contains a variety of immune cells, which play key roles in the initiation and progression of cancer (illustrated in Figure 1) (89, 90). Two immune-cell types in particular have been well characterized within the tumor microenvironment: T cells and tumor-associated macrophages (TAMs). TAMs can be separated into two phenotypes, the cytotoxic M1 phenotype (91) and the growth-promoting M2 TAMs that are involved in promoting tumor progression and are thought to correlate with poor prognosis in some settings (92). TAMs also modify the ECM and are involved in tumor recognition and antigen presentation (93). Moreover, they are important components of angiogenesis, invasion, and metastasis (94), and together with T helper (Th)-2 (95) and Th17 cells (96) can be involved in tumor promotion, progression, or metastasis. T cells are another important immune component of the tumor microenvironment. They can be categorized into different types, including the CD4^+^ T lymphocytes, which are further classified into Th cells (Th1, Th2, Th17), T-regulatory (T_reg_) cells, and the CD8^+^ cytotoxic T cells. Each of these cell types has an important role in supporting tumorigenesis. Tumor-localized CD8^+^ cytotoxic T cells are associated with indolent disease, while the presence of Th2, Th17, and T_reg_ polarized CD4^+^ T lymphocytes are linked to more aggressive disease. T_reg_ cells also have a key role in tumor immune evasion and angiogenesis (97, 98), and are negative prognostic indicators of overall survival in metastatic colon cancer (99). Both tumor-infiltrating lymphocytes and tumor-associated neutrophils are also of consequence in tumorigenesis. Tumor-infiltrating lymphocytes have been shown to regulate progression and subsequent metastasis in melanoma (100, 101), whereas tumor-associated neutrophils can either act as pro- or anti-tumorigenic depending on their polarization (102).

Clearly, the host immune system is important in controlling tumor progression and metastasis, and strategies that focus on targeting the immune system in cancer are gaining popularity. For example, monoclonal antibodies directed toward the anti-cytotoxic T-lymphocyte antigen 4 (CTLA-4), programed death 1 (PD-1), and PD-L1 inhibitory immune receptors causing immune checkpoint blockade have proven very successful in treating patients with advanced melanoma, where overall survival was improved and durable objective responses were observed (103). Adoptive T-cell immunotherapy strategies have also been successful. Sipuleucel-T is an autologous cellular immunotherapy that has been approved by the FDA for the treatment of asymptomatic or minimally symptomatic metastatic castration-resistant prostate cancer. This agent, which activates T cells causing them to target and attack prostate cancer cells, offered a survival advantage over standard clinical management (104); Sipuleucel-T is currently recommended as a first-line treatment in asymptomatic patients (105). More recent strategies, including one described by Brentjens et al. (106), which use chimeric antigen receptor T cells to recognize a predefined target by an antibody-derived binding domain, have proven successful in leukemia where persistent complete responses have been observed.

**Figure 4:**
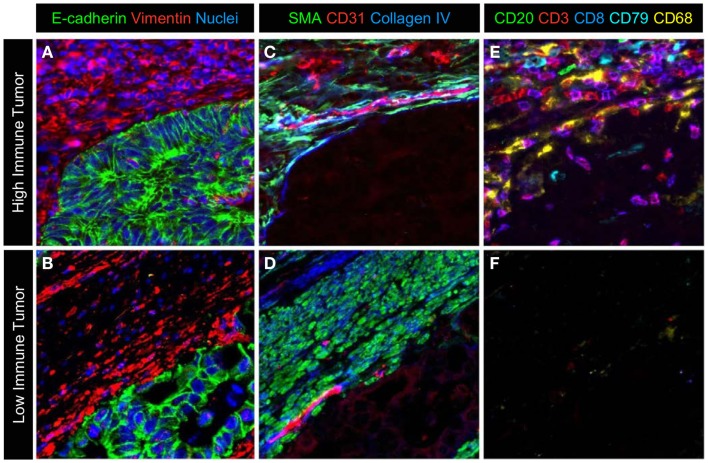
**A representative image of the different cell types in the microenvironment**. A multiplexed protocol on the MultiOmyx™ platform was used to generate an image that illustrates the differences in immune-cell infiltration seen in colorectal cancer. **(A,B)** Tumor cells and stromal cells are labeled with E-cadherin, vimentin, and nuclear counterstain. **(C,D)** Smooth muscle actin positive pericytes and smooth muscle and CD31^+^ endothelial cells together with extracellular matrix protein collagen IV identifies a subset of cells and ECM structure in the adjacent tumor microenvironment. **(E,F)** CD20^+^ and CD79^+^B lymphocytes, CD3^+^ and CD3^+^/CD8^+^ T-lymphocytes and CD68^+^ macrophages are present at high levels in the stroma and infiltrating the epithelium of the tumor in **(E)**, while sparse immune infiltration is seen in the tumor in **(F)**.

However, immune infiltrates are heterogeneous and differences in cell types and location need to be considered in any analysis approach (Figure 4). For example, the number, type, and location of tumor-infiltrating lymphocytes in primary tumors has been used to develop an “immunoscore” that has not only prognostic but also predictive value (107). One limitation of this approach is that multiple markers may need to be used to determine the exact immunoprofile of each patient. Enumeration and characterization of immune cells and their associated phenotypes in the tumor microenvironment using *in situ* multiplexed, analytical approaches should have utility in this situation. The MultiOmyx™ platform has recently been applied in the diagnosis of Hodgkin Lymphoma. A single slide multiplexing protocol that includes measurement of nine biomarkers (CD30, CD15, CD45, Pax5, CD20, CD79a, OCT2, Bob1, and CD3) was used to diagnose patients with classical Hodgkin Lymphoma with high sensitivity and specificity (108).

## Moving Technology Forward – What’s Next for Delineating Cancer Mechanisms and Identifying Diagnostic Biomarkers?

In addition to the previously discussed technologies, there are a number of other analytical methods used in cancer research, drug, and biomarker discovery process. As mentioned earlier, IHC is routinely used for measurement of protein expression in FFPE tissue, but chromogenic detection is primarily limited to single marker analysis with non-linear staining intensity. Since a new tissue section is required for each analyte, analysis of multiple proteins may be problematic if the tissue or tumor area is limited. In contrast, fluorescence-based imaging allows multiplexed analysis of up to seven proteins in a single sample and higher-order multiplexing (or hyperplexing) fluorescence imaging methods measure between 60 and 100 proteins in a single sample (71, 109). DNA FISH is a cytogenetic assay to determine copy number, gene loss, mutations, or rearrangements, and is commonly used in research and cancer diagnostics. MultiOmyx protein multiplexing platform also incorporates a DNA FISH measurement, thus allowing combined interrogation of genome and protein heterogeneity in a single sample (71).

Flow cytometry is a mature technology routinely used in research and in clinical practice for the multiplex analysis of hematological and non-solid tumors. It can simultaneously measure multiplexed-biomarker information on thousands of cells per second, and has been critical for defining different immune-cell populations. Phospho-flow cytometry has also been used to characterize cell signaling networks by measuring the phosphorylation patterns of proteins in individual cells (110–112). In addition to multi-parametric measurements, it has been adapted for sorting live cells into pure populations for subsequent analysis. Over the last five decades, flow cytometry capabilities have increased with the availability of new instruments such as the LSRFortessa^TM^ X-20 (BD Biosciences, San Jose, CA, USA), which is able to measure 20 fluorescent channels simultaneously. However, while flow cytometry can be used for tissue analysis after enzymatic dissociation, the process itself destroys any spatial aspect of expression patterns, which somewhat limits the utility of the technique for research. Additionally, tissue disaggregation methods invariably lead to cell damage and/or incomplete dissociation and may be limiting in the analysis of rare cell populations. IHC and multiplex *in situ* immunofluorescence techniques, on the other hand, maintain spatial context and information on each individual cell.

In recent years, use of next-generation sequencing (NGS) technologies for DNA, RNA, and epigenome analysis has dramatically risen due to decreased cost and technology access and maturity (113). While enabling unprecedented level of molecular analysis of samples there are some technical challenges and potential barriers to clinical adoption. For example, biopsy samples may provide an insufficient amount of tumor DNA; identification and validation of actionable mutations requires large clinical trials and complex bioinformatics tools (114). Formalin-fixed samples present several analytical challenges due to increased DNA fragmentation and chemical modifications caused by the fixation process (115, 116). Depending on the sample age and the extent of the fixation, sample quality can vary significantly and this can lead to errors when identifying mutations. This may be especially problematic for detecting rare somatic mutations in heterogeneous tumors. For FFPE samples with a low amount of usable input DNA, the problem of inadequate sensitivity is coupled with a larger number of false positives due to C to T transitions. RNA is even less stable than DNA and, furthermore, the quantity and the quality of the RNA isolated from FFPE tissues is usually inferior relative to that obtained from fresh tissue (117). In spite of this, optimized and standardized sample preparation can allow the retrieval of sufficient mRNA for expression analysis (118). Encouraging reports have shown good correlation between gene expression from FFPE specimens and fresh-frozen matched tissues or protein expression measured by IHC (119, 120).

Mass spectrometry is an increasingly common tool for the multiplexed analysis of tissues with utility in differentiating cancer cells from normal tissue, and in novel biomarker discovery. The method can be used to analyze a wide range of analytes (small drug metabolites to large proteins) and a wide range of sample types. New methods for sample preparation, ionization, and mass analysis have enhanced current methods while enabling new ones. These methods can be broadly categorized into three areas: homogenized samples; label-free mass spectrometry imaging (MSI); and labeled MSI. The initial use of MS for the analysis of tissue that also attains the highest degree of multiplexing is achieved through homogenizing the sample and analyzing it with the combination of liquid chromatography separations and tandem MS detection. This method has the capability of analyzing and quantifying thousands of analytes, including proteins, metabolites (121, 122), and lipids (123) from a single sample. For example, Wisniewski et al. analyzed the proteome of FFPE samples from colonic adenomas and identified more than 7500 proteins to relate their expression levels to disease state (124). Variations of the achieved mass signatures can be used for analysis of post-translational modifications and quantitation. The ability of this method to discriminate between many analytes present simultaneously, with varying post-translational modifications, and at different concentrations, makes this method most adept for biomarker discovery. The breadth of this capability does come at a cost; increased time is needed for sample preparation, protocol development, and data analysis. Additionally, spatial information for each analyte is also lost via homogenation.

Label-free MSI is emerging as an important tool for molecular profiling of intact tissue samples while preserving spatial information about their expression (125). Label-free MSI does not require prior knowledge of markers to be profiled and is able to readily differentiate post-translational modifications as well as different isoforms of proteins. Additionally, the technique is applicable to multiple target types including drugs, metabolites, lipids, peptides, proteins, and even nucleic acids. While an MSI method is generally optimized for one type of target (e.g., lipids), it has the potential to detect multiple species of that target simultaneously. One limitation of label-free MSI is that it can generally only be used for relative, not absolute, quantitation, and it produces large data sets that, depending upon the size of the data set, take weeks to analyze (125).

Labeled-MSI uses antibodies similar to traditional IHC for detecting specific proteins or molecules in a sample. In labeled-MSI, the antibodies are labeled with a metal isotope tag that can be detected by MS. One of the original versions of this, cytometry time-of-flight (CyTOF) MS, combined inductively coupled plasma with TOF MS and has now been optimized for the real-time detection of multiple biomarkers in single-cells present in suspension (126). Recently, this methodology has been extended to intact tissues, with a new technique known as “imaging mass cytometry.” Imaging mass cytometry combines high lateral resolution laser ablation (127) with CyTOF mass cytometry detection to obtain subcellular spatial information about multiple biomarkers on FFPE tissue sections (128). The method has resulted in images with the lateral resolution necessary for morphological assessment in cancer diagnostics and has the capacity to analyze over 100 biomarkers simultaneously. It is currently limited by the availability of metal-tagged antibodies; only 32 rare earth metals are available for antibody labeling (128). This method was improved upon to create multiplexed ion beam imaging (MIBI) where the metal-tagged antibodies are ionized by the higher spatial resolution method of Secondary Ion Mass Spectrometry (SIMS) (129). During MIBI analysis, the metal antibodies are liberated as secondary ions, which are then analyzed with a magnetic sector mass analyzer; other mass analyzers (such as TOF) could also be used. This technique has been used successfully in imaging breast tumors, where 10 different targets were analyzed simultaneously (129).

## Summary

As the insights into cancer biology have evolved, emphasis has shifted toward understanding cancer subtype, cell-to-cell interactions, signaling pathways, tumor microenvironment, and immune-mediated responses. A detailed mechanistic understanding of how individual mutations contribute to modifying gene expression and protein function is required, as is the elucidation of how the various regulatory and metabolic pathways interconnect. Novel multi-omic technologies, including those that are based on multiplexed imaging of intact tissue, NGS, MS analysis, and gene expression allow the collation of large amounts of information within cells and tissue, and highlight the mounting challenge of how to integrate and compare such data. Multi-omic analysis at the cell level provides a much deeper insight into cell changes, interactions, and progression to metastatic disease. The use of quantitative imaging technologies is essential for visualization of tumor and cell behavior, low abundance proteins, rare cell events, and spatial distribution of cells and proteins. Adopting a comprehensive multi-omic approach should ultimately facilitate the identification of biomarkers that have diagnostic and prognostic value, and help match patients to the most appropriate treatment strategy.

## Conflict of Interest Statement

The authors are employes of the General Electric Global Research Center, and funding support for this manuscript was provided by GE Healthcare.
